# Detection of Nuclear Sources by UAV Teleoperation Using a Visuo-Haptic Augmented Reality Interface

**DOI:** 10.3390/s17102234

**Published:** 2017-09-29

**Authors:** Jacopo Aleotti, Giorgio Micconi, Stefano Caselli, Giacomo Benassi, Nicola Zambelli, Manuele Bettelli, Andrea Zappettini

**Affiliations:** 1 Department of Engineering and Architecture, University of Parma, 43124 Parma, Italy; jacopo.aleotti@unipr.it (J.A.); micconi@ce.unipr.it (G.M.); caselli@ce.unipr.it (S.C.); 2 due2lab s.r.l., 43121 Parma, Italy; benassi@due2lab.com (G.B.); zambelli@due2lab.com (N.Z.); 3 IMEM-CNR, 43124 Parma, Italy; manuele.bettelli@imem.cnr.it

**Keywords:** CdZnTe-based detector, nuclear radiation detector, haptic teleoperation, unmanned aerial vehicles

## Abstract

A visuo-haptic augmented reality (VHAR) interface is presented enabling an operator to teleoperate an unmanned aerial vehicle (UAV) equipped with a custom CdZnTe-based spectroscopic gamma-ray detector in outdoor environments. The task is to localize nuclear radiation sources, whose location is unknown to the user, without the close exposure of the operator. The developed detector also enables identification of the localized nuclear sources. The aim of the VHAR interface is to increase the situation awareness of the operator. The user teleoperates the UAV using a 3DOF haptic device that provides an attractive force feedback around the location of the most intense detected radiation source. Moreover, a fixed camera on the ground observes the environment where the UAV is flying. A 3D augmented reality scene is displayed on a computer screen accessible to the operator. Multiple types of graphical overlays are shown, including sensor data acquired by the nuclear radiation detector, a virtual cursor that tracks the UAV and geographical information, such as buildings. Experiments performed in a real environment are reported using an intense nuclear source.

## 1. Introduction

UAVs in nuclear inspection tasks can be either teleoperated by a human operator, usually with standard remote controllers [1,2], or fly autonomously [3,4,5,6,7,8,9], following a predefined route or using an exploration behavior. In both cases, an expert human operator is required to oversee the entire operation, being aware of the state of the UAV, verifying sensor data acquisition and checking for potential obstacles or dangerous conditions related to the mission. The advantage of using human teleoperation is that an expert operator can focus on selected areas of the environment rather than calling for an exhaustive scan, thereby overcoming the problem of the limited duration of each flight afforded by onboard batteries. However, UAV teleoperation in critical tasks raises a fatigue issue: maintaining a high degree of situation awareness is challenging, as it imposes a high mental demand for the human operator. Hence, it is crucial to provide additional information to the pilot by exploiting multiple feedback channels. Potential applications are the localization and identification of radioactive materials in industrial plants (such as steel mills), construction, recycling factories and landfills.

This paper investigates visuo-haptic teleoperation of an unmanned aerial vehicle carrying a custom nuclear radiation detector for environmental monitoring. To maintain a high level of situation awareness, the VHAR interface provides visual feedback in real time and, simultaneously, force feedback through the haptic device to the operator. The use of visuo-haptic interfaces for UAV teleoperation has not been considered in previous works. In particular, a haptic rendering algorithm is presented, based on impedance control, that provides an attractive force feedback around the location of the most intense detected radiation source. The purpose of the attractive force feedback is to keep the UAV close to the nuclear radiation source once a target is found. Preliminary experiments with users [10] have shown that mental load and difficulty in associating perception to sensor localization increase as the UAV flies farther from the operator. Hence, in order to provide a more adequate support for critical operations, visual feedback is added, in addition to haptic feedback, to convey coherent information to the operator.

Visual feedback is provided to the user on a computer screen using augmented reality. The video stream of a fixed camera on the ground, which observes the environment where the UAV is flying, is augmented with graphical overlays. In particular, a 3D histogram of the measured radiation intensity is displayed on top of the video stream to let the operator see the most recent measured intensity values, as well as the location of the maximum radiation found during the mission. A 2D virtual cursor is also displayed around the UAV that is computed from a vision-based tracking algorithm. Visual tracking not only facilitates the operator to detect the UAV on the image when flying at large distances, but it also improves the estimation of the UAV 3D pose and, therefore, the localization of the nuclear sources, compared to the accuracy that would result by using the UAV onboard sensors alone. Other elements such as buildings in close proximity to the UAV are retrieved from a geographic information system (GIS), registered using a semi-automatic approach and highlighted on the screen. Finally, simple bounding boxes of the building are used to generate geo-fences for the UAV, i.e., a virtual perimeter for collision avoidance.

**Figure 1:**
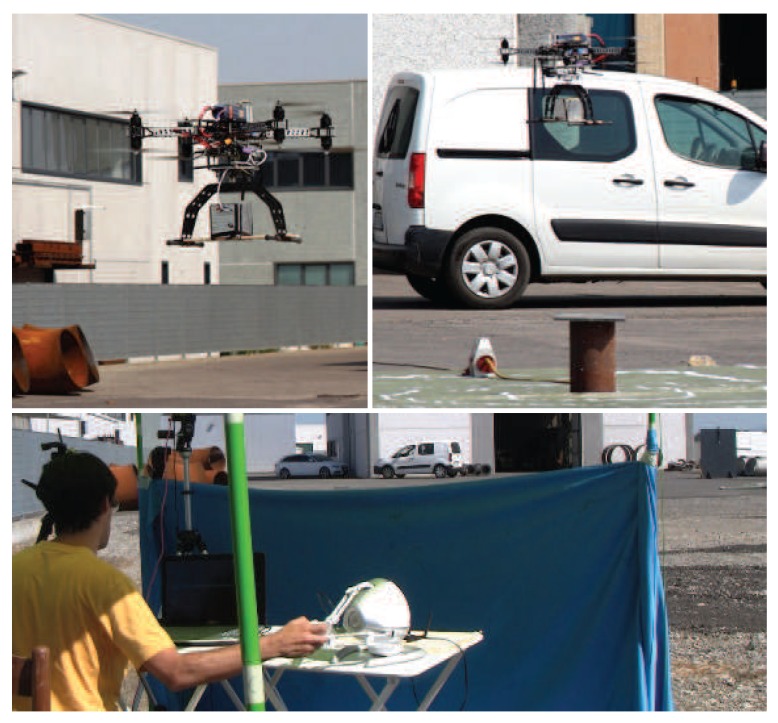
UAV equipped with a CdZnTe gamma-ray detector in flying tests (top). The operator using the visuo-haptic user interface (bottom). The operator sits in front of a cloth that prevents him from seeing the nuclear source on the ground.

Complete experiments in a real environment have been performed with an intense nuclear source as shown in Figure 1, under the supervision of the public environmental protection agency. Experiments have been performed by an expert operator due to the critical setup. The UAV was clearly visible to the operator, but the location of the radiating substance was not known to the operator in advance. Quantitative data have been collected such as the task completion time and the error between the location of the radiating substance, estimated by the operator, with respect to its actual location taking advantage of a video camera mounted on the UAV. Experiments show that a teleoperation approach that supports switching between heading-based and position to position control modes increases the position detection accuracy of the radio-active material with respect to a pure heading-based control mode [10,11]. Usability experiments, performed in a simulated environment, are also reported. Results indicate that adding visual feedback does not further improve position detection accuracy, but it increases the situation awareness of the operator and reduces mental workload.

Small multi-rotor unmanned aerial systems can obtain high spatial resolution maps of radiological contamination sources as pointed out in [2]. Several authors have investigated the application of UAVs for monitoring environmental radioactivity [1,2,3,4,5,7,9,12,13,14]. However, none of these works has considered the use of haptic teleoperation for nuclear radiation detection. Indeed, either standard remote controllers were adopted or pre-programmed flight missions were used for autonomous UAVs. A survey of networking aspects for small unmanned aerial systems is reported in [15].

In [12] a UAV-mounted biosensor system is described for environmental monitoring applications including radiation leakage detection. Okuyama et al. [9] developed an autonomous helicopter for measuring radiation data during a flight, with real-time data transmission including images to a monitoring ground station. Boudergui et al. [1] provided a preliminary evaluation of a teleoperated UAV equipped with a CdZnTe sensor and a gamma camera for nuclear and radiological risk characterization. However, the system was developed for indoor environments, whereas we focus on outdoor environments, which pose different problems in terms of UAV localization, as well as the operator’s situational awareness. Unmanned aerial vehicles with a fixed wing, flying at high altitude and high speed have been presented in [5,13] for radiation detection in outdoor environments. In [7], a remotely-piloted UAV was proposed to measure hazardous gaseous sources. Martin et al. [3] presented a UAV for the radiological characterization of uranium mines. Sanada et al. [14] developed an unmanned helicopter to monitor radiation at the Fukushima Dai-ichi nuclear power plant (FDNPP). Radioactive cesium deposition was successfully measured on the ground. In [4], an unmanned aerial system was presented that was capable of producing more accurate radiation distribution maps in the FDNPP with a resolution of more than 1 m. In other works [6,16], simulation results have been reported. In [16], a simulated UAV for imaging and radiation detection was developed using an autonomous helicopter. In [6], a simulation was developed with multiple UAVs for contour mapping of nuclear radiation with formation flight control.

In previous works, augmented reality for unmanned aerial vehicles has been investigated mainly by using videos from onboard cameras. In [17], a UAV equipped with a video camera was used to generate an augmented reality environment for construction site monitoring that supported registration and visualization of 3D building models. In [18], a similar approach for real-time UAV video augmentation was presented with applications to disaster response. In [19], another augmented telepresence system was developed for large-scale environments by exploiting an omni-directional camera. Iwaneczko et al. [20] presented a heads-up display to be used in UAV ground control stations to improve the UAV manual control performance of the operator. In [21], a mixed reality environment was developed where a user could interactively control a UAV and visualize range data in real-time. The closest work to ours using a fixed ground camera was proposed by Zollmann et al. [22], where an augmented reality system was developed. However, the system was aimed at specifying waypoints for the UAV from a touchscreen and at checking for potential collisions with the surrounding environment.

So far, haptic teleoperation of aerial vehicles has been investigated exclusively for collision avoidance or to make the flight process easier [23,24,25,26,27,28,29,30,31,32,33,34,35,36]. Reyes et al. [23] developed a remotely-operated UAV for indoor and outdoor environments where force feedback is proportional to the translational speed and proximity to objects. In [24], a novel intuitive technique for UAV teleoperation was introduced where a repelling force feedback is generated proportional to the UAV’s velocity. Lam et al. [25] investigated artificial force fields to generate haptic feedback in UAV teleoperation in simulated scenarios. In [26], an approach was presented for target identification and obstacle avoidance in indoor environments. A bilateral control system was developed for haptic teleoperation with force feedback, and a 3D map of the environment was built using computer vision. In [27], an intuitive teleoperation technique was presented, including force feedback, to safely operate a UAV by an untrained user in cluttered environments. Masone et al. [28] proposed a method for semi-autonomous UAV path specification and correction where a human operator could modify the shape of the path of the UAV, while an autonomous algorithm ensured obstacle avoidance and generated force feedback. In [29,30], an admittance haptic control technique was introduced based on both the UAV position, which was tracked by an indoor visual sensor, and on the force exerted on the haptic device. Ruesch et al. [32] proposed a UAV haptic teleoperation method to overcome the workspace limitation of the haptic device and cover large distances.

CdZnTe detectors, thanks to their wide operative energy range, room temperature operation and good spectroscopic properties, have been used in many fields such as in medical applications [37], security [38], environmental control [39] and astrophysics [37]. Furthermore, CdZnTe detectors are of particular interest in UAV applications due to their high stopping power for high energy gamma radiation, robustness, low weight and low power consumption.

The paper is organized as follows. Section 2 provides an overview of the system. Section 3 describes the components of the visuo-haptic augmented reality interface. Section 4 presents the experimental results. Section 5 concludes the paper, discussing the results and providing suggestions for possible extensions.

## 2. Overview of the System

**Figure 2:**
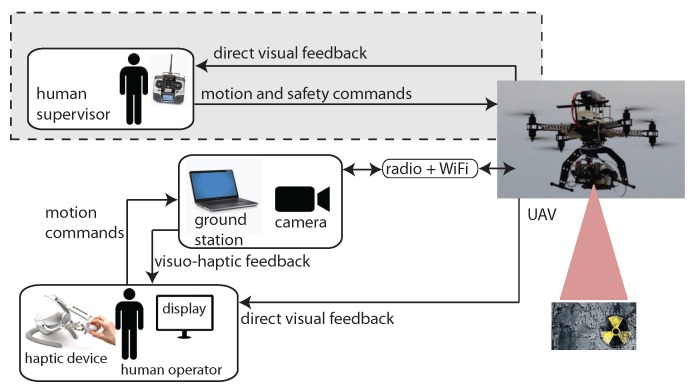
Overall architecture of the system.

Figure 2 shows the overall architecture of the proposed system, which is based on the ROS framework. In nuclear source detection tasks, the UAV flies at a constant altitude and constant speed. A low velocity set point value is adopted since a high speed would affect georeferencing of the sensor data. The operator of the haptic device is in charge of guiding the UAV during the hazard search and localization phase by sending motion commands on the xy plane (parallel to the ground) while receiving visuo-haptic feedback. A fixed camera on the ground observes the UAV and provides visual feedback. The camera is connected to the ground station through a Gigabit Ethernet cable. Visual feedback is displayed on a computer screen while a 2D planar force feedback is conveyed through the haptic device connected to a ground station. A second human operator (supervisor), using a standard remote controller, is responsible for take off, landing and setting the altitude set point. The second operator can also take full control of the UAV at any time, as required by the Italian regulation for unmanned air vehicles, thus overriding haptic commands. Both operators have direct sight of the UAV. The main functions of the ground station are to process motion commands provided by the operator of the haptic device, to send commands to the UAV, to receive sensor data from the UAV, and to compute and render both visual and force feedback. Information received by the ground station consists of UAV telemetry data (including position, speed, height and battery charge), sent through the UAV radio link, and sensor data from the onboard gamma-ray detector (number of photon counts for each energy band in a fixed time frame), sent through a dedicated wireless link.

Assuming a planar environment, the gamma-ray detector will measure a maximum intensity when it is on the vertical of the radiating target. The UAV GPS coordinates will be assumed as the coordinates of the radiating part at the ground. The haptic device used in this work is a 3DOF Novint Falcon, with a resolution of about 400 dpi, a maximum force feedback capability of about 10 N and a range of motion of about 10 cm${}^{3}$.

### 2.1. UAV Platform

**Figure 3:**
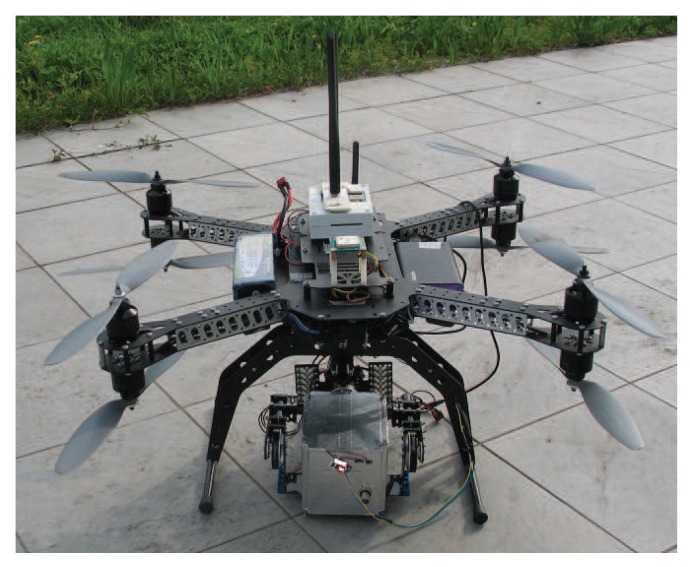
UAV equipped with the CdZnTe gamma-ray detector (mounted at the bottom).

The UAV equipped with the CdZnTe gamma-ray detector is shown in Figure 3. The aerial vehicle is an octocopter in coaxial configuration, produced by Virtual Robotix Italia, with a gross payload up to 4 kg and a maximum flight time of about 10–15 min. The UAV transmits telemetry data to the ground station (868 MHz RF link). The UAV is built with a mixed Carbon and Aluminium structure. The size of the frame is within 550 mm (without propellers). The UAV is equipped with an MEMS accelerometer, gyro, magnetometer and GPS sensors. A VRBrain autopilot system is used (based on the ArduCopter firmware adapted by Virtual Robotix Italia), which comprises a 168-MHz ARM CortexM4F microcontroller with DSP and floating-point hardware acceleration. The autopilot system supports multiple flying modes such as loiter, return to home and guided mode.

The CdZnTe gamma-ray detector is enclosed in a box and mounted on a two-axis brushless gimbal. An embedded system based on the Intel Galileo board reads sensor data from the gamma-ray detector and sends the data stream to the ground station through a dedicated long-range 5-GHz WiFi connection. Intel Galileo is a single core i586 platform (400 MHz, 256 MB RAM). The dedicated wireless data connection link avoids bandwidth contention on the UAV RF channel and does not affect the UAV autopilot system, which runs on a real-time operating system. Two external antennas are connected to the embedded platform allowing a WiFi communication range up to 170 m. The embedded system is powered by an external Li-Ion 10-Ah, 5-V battery pack.

### 2.2. CdZnTe Gamma-Ray Detector

The goal of the gamma-ray detector is to detect nuclear sources on the ground in a wide energy range to reveal the most dangerous contaminants that may be dispersed in the environment. The detector, designed and developed for this work, is lightweight (about 0.3 kg), and it has low power consumption. The measurable energy range is from 10 KeV–1.5 MeV so that all o the main nuclear isotopes can be detected. Radioactivity data measured by the detector are represented by a histogram of 2800 energy bands. Each bin *i* contains the number of counts C[i] detected in a time frame ▵t=2 s. The count rate *C* for each energy band *i* varies according to the inverse square law:

(1) $$C\propto \frac{1}{l^{2}}$$

where *l* is the distance to the nuclear source. The time required to transmit a full spectrum to the ground station, through the WiFi link, with all 2800 energy bands (including sensor reading and transmission) is about 2.2 s (0.45 Hz). The sensor features a large field of view. Since the higher the energy of the photon to be revealed, the larger the thickness of the detector that must be used, a 6 mm-thick CdZnTe (cadmium zinc telluride) detector was adopted. As one of the most important applications of the proposed system is the detection of nuclear sources considered dangerous for operators and workers in industrial plants, the detector was designed to measure nuclear sources whose average effective dose is 1 mSv/year at a 1-m distance. Indeed, 1 mSv/year is the dose limit set by the law for workers. Table 1 reports the typical number of counts per second measured by a 20×20×6 mm detector at a 2-m distance from some nuclear sources. The values indicate that by using the proposed detector at about 2 m from the ground, it is possible to measure a number of counts per second that is sufficient for localizing nuclear sources that are relevant for workers’ safety. The choice of a single carrier device was due to the fact that hole transport properties are not good enough to ensure optimal charge drift over several millimeters. Thus, the detector was fabricated by using a contact geometry known as “drift strip”, which is known to ensure good energy resolution even for large volume detectors [40].

**Figure 4:**
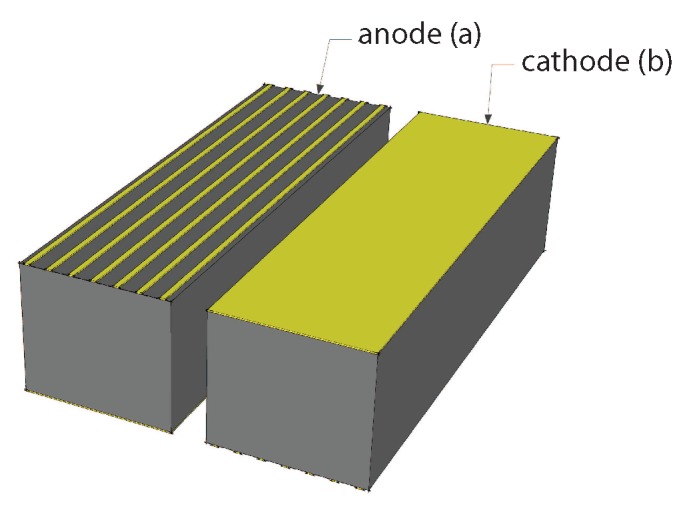
Drift strip gamma ray detector: anode (**a**) and cathode (**b**).

**Figure 5:**
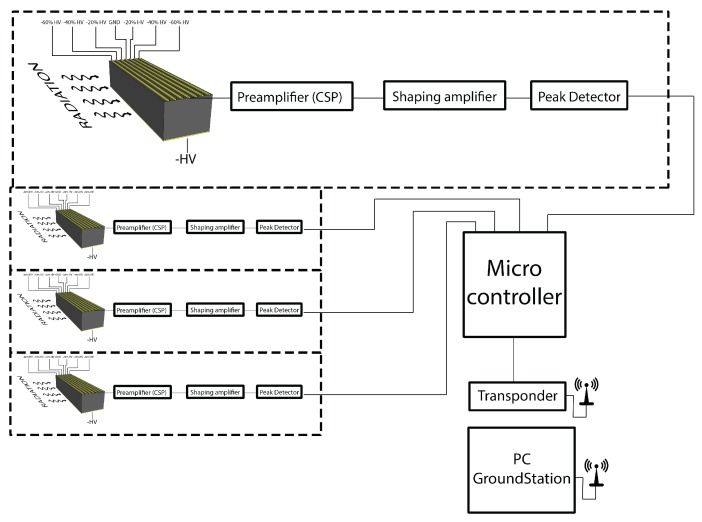
Read out electronic, with charge sensitive preamplifier (CSP), and data transmission scheme.

Four 5×6×20 mm^3^ drift strip detectors were realized using CdZnTe material acquired from Redlen. Figure 4 shows the contact geometry of the anode and cathode contacts. The anode is realized with seven strips, the central being the collecting one, the adjacent strips are polarized in such a way to drift the carriers towards the collecting strip. Electroless gold contacts were used in order to obtain blocking contacts [41], and surface passivation was adopted to reduce the surface leakage current. Analog electronics were used to read out the generated signals, according to the scheme in Figure 5. The detector, the read out electronics, the batteries and the DC-DC circuit, which generates the high voltage supply, were stored in a box easily mounted on the UAV, as shown in Figure 3.

An automatic procedure is performed at the beginning of each flight for background radiation removal (the sensor does not have a built-in auto-calibration feature) assuming that the UAV take off location is far away from all of the nuclear sources of the environment. Indeed, once the UAV is airborne and hovering at the desired height, a set of radiation measurements is taken for some time frames $▵t_{j}$, j=1…K, then the intensity of the background radiation is set as $I_{b}=\max_{▵t_{j}}\sum_{i}C_{b}\left[i\right]$, i.e., the largest measured value of the sum of the counts over all energy bands i=1…2800. Then, when the UAV is flying during the teleoperated mission, the current radiation intensity *I* in a time frame is georeferenced using the current estimated UAV pose, and it is used to update the position on the ground with the highest radiation that in turn is needed to generate force feedback (as explained in Section 3.1). The current radiation intensity is computed as the difference between the current measured value $\sum_{i}C_{m}\left[i\right]$ and the background radiation acquired at the beginning of the mission, i.e.,

(2) $$I=\left\{\begin{array}{cc} \sum_{i}C_{m}\left[i\right]-I_{b}-\gamma & \mathrm{if}\;\left(\sum_{i}C_{m}\left[i\right]-I_{b}\right)>\gamma ,\\ 0 & \mathrm{otherwise}\;, \end{array}\right.$$

where γ is a small threshold. The proposed approach for background radiation removal is more realistic than the one proposed in [10], which was adopted for the initial evaluation of the teleoperation system in experiments with simulated nuclear radiation sources.

## 3. Visuo-Haptic Augmented Reality Interface

### 3.1. Haptic Teleoperation

**Figure 6:**
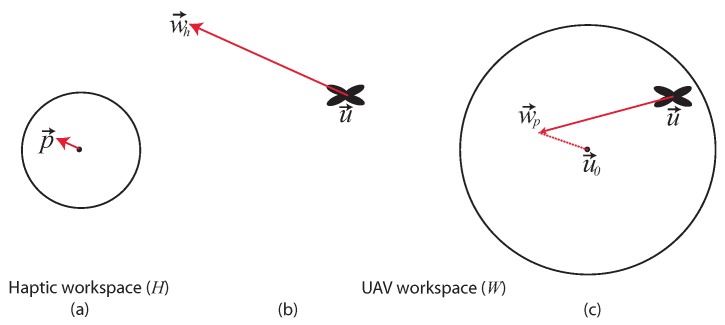
Diagram illustrating the two haptic teleoperation techniques. Haptic workspace on the x,y plane (**a**). Heading-based control technique (**b**); the current displacement $\vec{p}$ of the haptic device is used to compute a heading direction with respect to the current UAV position. Position to position control technique (**c**); $\vec{p}$ is used to compute a waypoint with respect to the center of operation $\vec{u_{0}}$.

Two impedance control modes (Figure 6) have been developed where the operator sends motion commands (via the haptic device tool point) and receives force feedback limited to the xy plane. Since inspection tasks require precise position-controlled flights, in both teleoperation modes, there is no direct mapping from the haptic device to the velocity of the UAV. Moreover, the two teleoperation modes have been designed to map the limited workspace of the haptic device to an arbitrarily large UAV workspace. In the first teleoperation mode ($M_{heading}$), position information read by the haptic interface (namely, the x,y coordinates of the tool point) is used to compute the horizontal heading direction of the UAV [10,11]. In particular, the current displacement $\vec{p}=\left(p_{x},p_{y},p_{z}\right)$ of the tool point of the haptic device with respect to its center is converted to a waypoint $\vec{w_{h}}=\left(x,y,z\right)$ for the UAV in world coordinates as follows:

(3) wh→=η∥p→∥00−11000−10︸THWα000α0000pxpypz+uxuyuz

(4) $$\eta =\left\{\begin{array}{cc} 0 & \mathrm{if}\;∥\vec{p}∥<D\\ 1 & \mathrm{otherwise} \end{array}\right.$$

where $\vec{u}=\left(u_{x},u_{y},u_{z}\right)$ is the current position of the UAV in the world reference frame, α is a constant and THW is the rotation matrix from the haptic coordinate system *H* to the world reference frame *W*. The world reference frame corresponds to the UAV pose at take off. A flat Earth model is assumed for the conversion between Cartesian and GPS coordinates. The UAV flies in guided mode following the current waypoint with yaw heading pointing towards the waypoint itself. Each waypoint $\vec{w_{h}}$ is not meant to be reached by the UAV as it is continuously updated and placed at a long distance from the current UAV position (α≫0), thus resulting in a heading-based approach. In Equation (3), the altitude of the generated waypoint (*z* coordinate) does not depend on the displacement of the tool point of the haptic device $p_{z}$. Indeed, as explained above, the UAV altitude set point $u_{z}$ is provided by the second operator via the standard remote controller or assumed as a default mission parameter. If the tool point of the haptic device is within a fixed range *D* from the center of the haptic reference frame, the waypoint is not updated (η=0 in Equation (4)), and the UAV hovers.

The second teleoperation mode is a hybrid approach that allows the operator to switch between the heading-based technique ($M_{heading}$), discussed above, and a position to position control technique ($M_{position}$). Mode switching is achieved by pressing one of the buttons of the haptic device. The position to position control technique, inspired by the approach proposed in [27], enables a direct mapping between the haptic device to the UAV position around a fixed center of operation. In particular, in this second teleoperation mode, a waypoint $\vec{w_{p}}=\left(x,y,z\right)$ for the UAV in world coordinates is computed as follows:

(5) wp→=η∥p→∥THWδ000δ0000pxpypz+u0xu0yu0zifMposition,wh→ifMheading.

where $\vec{u_{0}}=\left(u_{0x},u_{0y},u_{0z}\right)$ is the center of operation, defined as the position of the UAV when $M_{position}$ is activated, and δ≪α is a constant. The idea is that the heading-based control technique can be used for long transition motions, e.g., when the UAV has to be moved to explore different regions of the environment, while the position to position control technique is helpful when the UAV explores an area in close proximity of a nuclear source. Although it is known that mode switching teleoperation can lead to instabilities, the proposed approach did not exhibit such a problem in any of the reported experiments (that included a single nuclear source), as well as in the simulated experiments, due to the low switching frequency (one to three mode switches per mission on average).

A force feedback $\vec{f}$ is provided to the operator, acting as a basin of attraction, to let the UAV fly close to the region where the radiation is maximal. Indeed, as the remotely-operated UAV travels away from the location of the most intense perceived radiation, the haptic device exerts a force in the horizontal direction towards the center of the haptic workspace. The force feedback is computed as:

(6) f→=ζ∥r→−u→∥01000−1−100︸TWHβ000β0000rx−uxry−uyrz−uz

(7) $$\beta =\frac{f_{max}}{L^{2}}d^{2}{\left(\vec{r},\vec{u}\right)}_{xy}$$

(8) $$\zeta =\left\{\begin{array}{cc} 0 & \mathrm{if}\;d{\left(\vec{r},\vec{u}\right)}_{xy}>L\\ 1 & \mathrm{otherwise} \end{array}\right.$$

where $\vec{r}=\left(r_{x},r_{y},r_{z}\right)$ is the estimated position of maximum radiation discovered so far by the UAV, which is updated periodically (with period ▵t). Radiation intensity *I* is computed using Equation (2). If the latest measured radiation intensity is greater than the maximum radiation found so far, the latest radiation intensity is set as the current maximum radiation. The force feedback has a quadratic profile (Equation (7)), where $d{\left(\vec{r},\vec{u}\right)}_{xy}$ is the distance between the projection of the UAV on the ground and $\vec{r}$, $f_{max}$ is the maximum exerted force and *L* is the radius of the basin of attraction. No force feedback is provided outside the basin of attraction (ζ=0 in Equation (8)). The provided force does not overpower the user who can also guide the UAV outside the basin of attraction if he/she desires. Moreover, the user can reset force feedback to zero by pressing a button on the tool point of the haptic device. This feature can be useful when multiple concentrated nuclear sources have to be explored.

### 3.2. Vision-Based UAV Detection

**Figure 7:**
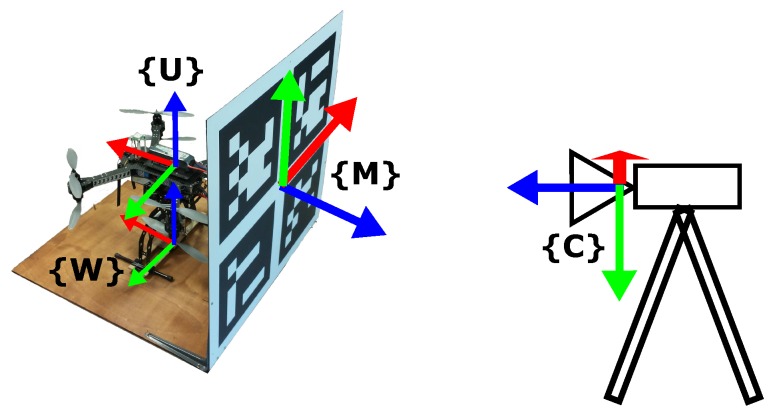
UAV takeoff platform with marker (80×80 cm) and reference frames.

A fixed camera on the ground observes the UAV work space including the takeoff area. A takeoff platform, shown in Figure 7, includes a marker that is used to calibrate the reference frames.

The current 3D position ${}^{C}u_{ekf}^{t}$ of the UAV in the camera reference frame *C* is given by ${}^{C}u_{ekf}^{t}={}_{M}^{C}T{}_{W}^{M}T\cdot {}^{W}u_{ekf}^{t}$, where ${}^{W}u_{ekf}^{t}$ is the current 3D position of the UAV in the world reference frame *W* (located at the base of the takeoff platform) provided by the autopilot extended Kalman filter (EKF). The EKF fuses data from the GPS sensor, IMU and barometric altimeter, and therefore, it is strongly affected by the position error of the GPS receiver. ${}_{W}^{M}T$ is a fixed transformation between *W* and the marker reference frame *M*, and ${}_{M}^{C}T$ is the transformation of the marker as seen from the camera reference frame.

The proposed approach for vision-based UAV detection, illustrated in Algorithm 1, exploits motion segmentation and SVM-classification using SURF local descriptors. The algorithm was developed on the OpenCV library. Main input data are the intrinsic camera parameters, the current image *S* from the camera, the UAV position estimated by the EKF in camera coordinates ${}^{C}u_{ekf}^{t}$, the projection of the UAV position on *S* at previous frame ${\bar{u}}_{ekf}^{t-1}$, a search window SW (defined by a center pixel and a size) and the last UAV predicted state ${\bar{u}}^{t-1}$ on *S*. The output is the current estimated position of the UAV in camera coordinates ${}^{C}u_{v}^{t}$. The estimated position of the UAV using visual detection is more accurate than the one provided by the autopilot EKF, and therefore, it also improves georeferencing of the nuclear source.

After projecting ${}^{C}u_{ekf}^{t}$ on the image plane (line 1), the foreground images F(S), representing moving objects, are computed (line 2) using a background subtraction algorithm [42]. A dilation is then applied to F(S) (line 3). An efficient search of the UAV is then performed in a sub-window SW of the dilated image $\bar{F}\left(S\right)$, centered at ${\bar{u}}_{ekf}^{t}$ (Lines 4–16). In particular, blobs are extracted from the sub-window, and then, the axis-aligned bounding box (AABB) $Box_{i}$ of each blob is computed.

Afterwards, each box is evaluated by a binary bag-of-words SVM classifier, trained to detect the UAV in the image, using SURF features (Lines 9–16). The box with the highest positive score BestBox, if any, is selected as the candidate AABB that contains the UAV (this case is named SURF). The 3D position of the UAV in camera coordinates ${}^{C}u_{v}^{t}$ is estimated by back projection of the center pixel of BestBox using the *z* coordinate of the orthogonal projection of ${}^{C}u_{ekf}^{t}$ on the optical ray (line 18). The current state ${\bar{u}}^{t}$ of the UAV on *S* is also updated using the BestBox center (line 19). Finally, the size of SW is updated to the size of BestBox enlarged by a constant value (line 20).

If a valid box is not found from the SVM classifier, the $Box_{i}$ having the largest intersection with the search window and whose distance to the predicted position ${\tilde{u}}^{t}$ of the UAV does not exceed the threshold value Th (Lines 22–30) is selected (this case is named the closest box). Then, the box center is used to compute the 3D position of the UAV in camera coordinates (line 32) as above. Prediction (line 22) is computed using an alpha-beta filter. The size of SW is also incremented by a small value (line 34). If a valid box is still not found, prediction ${\tilde{u}}^{t}$ is used to compute the 3D position of the UAV in camera coordinates (line 36), and ${\bar{u}}^{t}$ is updated using the difference vector between the last two measured positions provided by the EKF (line 37) (this case is named prediction). Since the last case is valid for short-term prediction only, after a fixed time frame, the 3D position of the UAV is directly set as ${}^{C}u_{ekf}^{t}$.

 **Algorithm 1:** Vision-based UAV detection.  **Input:** Intrinsic camera parameters   *S*: Image at *t*   ${}^{C}u_{ekf}^{t}$: UAV position at *t* from EKF in camera frame   ${\bar{u}}_{ekf}^{t-1}$: UAV position from EKF at t−1 on *S*   SW: search window (center, size)   Th: displacement threshold between two frames   ${\bar{u}}^{t-1}$: last UAV predicted state on *S*  **Output:**
${}^{C}u_{v}^{t}$: estimated UAV position    1: ${\bar{u}}_{ekf}^{t}\leftarrow$
${}^{C}u_{ekf}^{t}$ projection on *S*    2: F(S)← Compute foreground image    3: $\bar{F}\left(S\right)\leftarrow$
F(S) dilation    4: $SW\leftarrow updateCenter(SW,{\bar{u}}_{ekf}^{t})$    5: C← Extract blobs in $SW(\bar{F}\left(S\right))$    6: **for**
$C_{i}\in C$
**do**    7:   $Box_{i}\leftarrow$ Extract AABB of $C_{i}$    8: **end for**    9: $Score_{max}\leftarrow 0$  10: **for each**
$Box_{i}$
**do**  11:   $Score_{i}\leftarrow$ Classify $Box_{i}$ with SVM  12:   **if**
$Score_{i}\geq Score_{max}$
**then**  13:     $BestBox\leftarrow Box_{i}$  14:     $Score_{max}\leftarrow Score_{i}$  15:     **end if**  16: **end for**  17: **if**
$Score_{max}>0$
**then**  18:     ${}^{C}u_{v}^{t}\leftarrow$
Backproject(center(BestBox))  19:     ${\bar{u}}^{t}\leftarrow center\left(BestBox\right)$  20:     SW←updateSize(SW,size(BestBox))  21: **else**  22:     ${\tilde{u}}^{t}\leftarrow predict\left({\bar{u}}^{t-1}\right)$ predicted position on *S*  23:     $Area_{max}\leftarrow 0$  24:     **for each**
$Box_{i}$
**do**  25:      $Area_{i}\leftarrow Box_{i}\cap SW$  26:      **if**
$Area_{i}\geq Area_{max}\wedge$          ${∥center\left(Box_{i}\right)-\tilde{u}∥}^{t}\leq Th$
**then**  27:        $BestBox\leftarrow Box_{i}$  28:        $Area_{max}\leftarrow Area_{i}$  29:      **end if**  30:     **end for**  31:     **if**
$Area_{max}>0$
**then**  32:      ${}^{C}u_{v}^{t}\leftarrow$
Backproject(center(BestBox))  33:      ${\bar{u}}^{t}\leftarrow center\left(BestBox\right)$  34:      SW←incrementSize(SW)  35:     **else**  36:      ${}^{C}u_{v}^{t}\leftarrow$
$Backproject({\tilde{u}}^{t})$  37:      ${\bar{u}}^{t}\leftarrow {\bar{u}}^{t-1}+{\bar{u}}_{ekf}^{t}-{\bar{u}}_{ekf}^{t-1}$  38:      SW←incrementSize(SW)  39:     **end if**  40: **end if**

### 3.3. Visual Feedback Displayed in Augmented Reality

**Figure 8:**
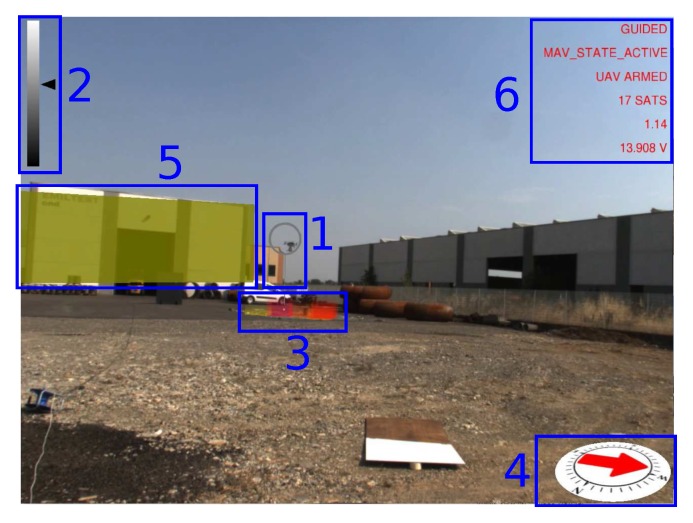
Augmented reality scene example with graphical overlays highlighted by blue boxes (for better visibility).

An augmented reality environment is generated and displayed on a computer screen to provide real-time visual feedback to the operator during the mission. 2D and 3D graphical overlays, called widgets, are rendered using the OpenGL library on top of the video stream acquired by the video camera. Geometric registration of the virtual camera is performed thanks to the calibration parameters obtained as explained in Section 3.2. An example of the augmented reality scene is shown in Figure 8.

Widget 1 is a virtual cursor displayed as a ring around the UAV that is computed thanks to the vision-based UAV detection algorithm. The virtual cursor tracks the UAV, and therefore, it helps the operator to quickly identify its current position. A vertical line is also displayed to show the current projection of the UAV on the ground. The ring is displayed on a gray scale according to the distance of the UAV to the camera. This information is also displayed by a 2D cursor moving on a vertical bar (Widget 2, top left) that acts as a distance-color map. The maximum distance (white color) in the experiments was set to 50 m.

Widget 3 is a colored 3D histogram that represents the distribution of the georeferenced radiation intensity *I* on the ground. The histogram shows the bin of the maximum detected radiation intensity during the mission and the closest k=25 bins to the current UAV position. The higher the bin, the higher the radiation intensity inside a cell of the grid. The highest bin has a fixed height, and all other bins are scaled in real time. The grid has a 1 m × 1 m cell size. The color of each bin also changes according to the radiation intensity value, ranging from green (low radiation intensity) to red (high radiation intensity).

The current UAV heading and magnetic north are displayed on a virtual compass (Widget 4). Geographical features in close proximity to the UAV workspace are retrieved from a query to the Open Street Map (OSM) database, registered in the augmented reality scene and highlighted on the screen (Widget 5) when the UAV flies close to them. Each building is displayed by computing an oriented bounding box from a 2D polyline provided by the geographical data. Buildings are also used to generate geo-fences for the UAV, i.e., a virtual perimeter for collision avoidance. Automatic registration only provides a coarse alignment of the geographical elements due to calibration errors and imprecisions in the OSM data. Hence, registration is manually refined during the calibration phase. More advanced approaches for automatic registration of buildings could be adopted [43,44,45]. However, these methods are beyond the scope of this work. Buildings are displayed using yellow color. Since OSM does not provide information about buildings’ height, the height is fixed to a constant value. The last graphical overlay (Widget 6) provides textual information to the operator about mission-critical parameters including current flight mode, flight status, horizontal dilution of precision (HDOP) and number of satellites currently visible.

## 4. Experiments

### 4.1. Preliminary Evaluation

**Figure 9:**
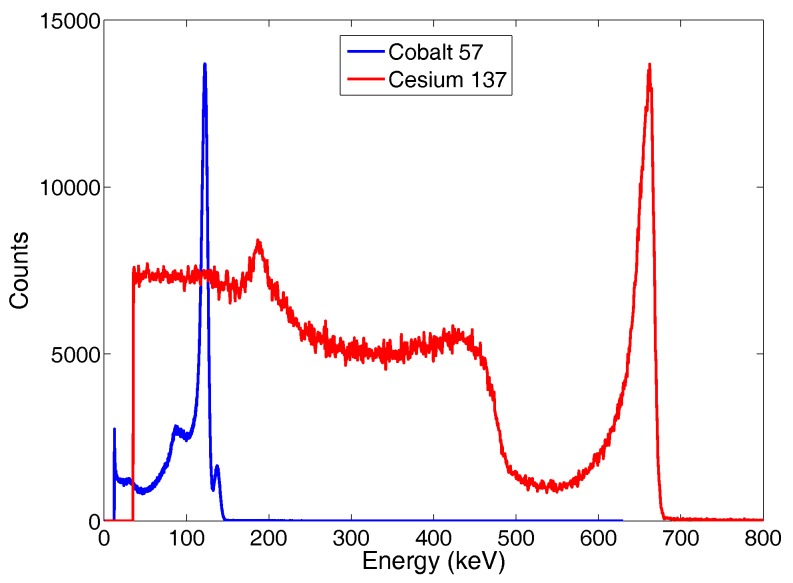
^137^Cs and ^57^Co emission spectra as measured by the detector and the electronic mounted on the UAV in an indoor laboratory setup.

Initial evaluation of the CdZnTe detector was carried out in an indoor laboratory setup by measuring the number of counts at each energy band for low intensity nuclear sources. Figure 9 shows the spectra obtained with ^57^Co and ^137^Cs nuclear sources. The energy resolution is 4% at 122 keV and 2.9% at 662 keV, in spite of the mechanical vibration induced by the UAV.

### 4.2. Evaluation of Nuclear Source Detection Accuracy in Real Environments

The visuo-haptic teleoperation system has been validated in an outdoor environment with a real nuclear source under the supervision of the public environmental protection agency. Experiments have been performed in daylight conditions with an intense ^192^Ir nuclear source located in a service area of an industrial plant located in Gossolengo, Piacenza, Italy. Performing experiments with real nuclear sources in operational environments is a complex endeavor, requiring formal authorizations. Due to this procedural complexity, it took us about one year to set up the experiment reported in this section. The nuclear source was put into a vertical lead container placed on the ground to prevent lateral radiation emission (Figure 3). Due to the high-intensity source, the container was closed at the top by a 3 cm-thick lead plate. The operator was kept at a safety distance of about 30 m from the nuclear source. The UAV maintained a height from the ground ranging from 1.5–3 m.

**Figure 10:**
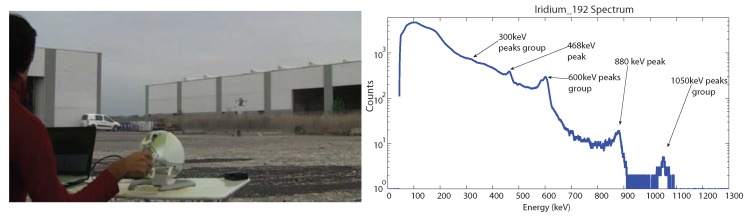
Detection of ^192^Ir in a real environment with heading-based teleoperation mode (the operator has direct sight of the nuclear source): image during the exploration task (left). Total radiation spectrum obtained by summing up all measurements (right). A video of the experiment is available as Supplementary Material (video1).

**Figure 11:**
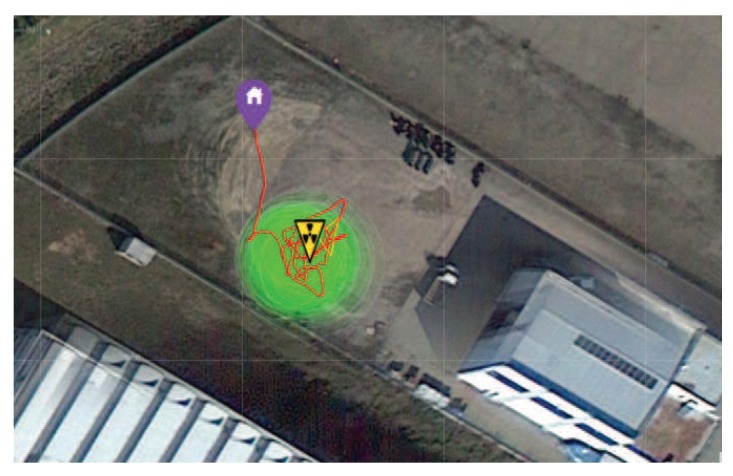
Detection of ^192^Ir in the real environment (the operator has direct sight of the nuclear source): flight path of the experiment shown in Figure 10; regions where radiation was measured by the CdZnTe detector (green circles with different sizes and shading) and the estimated location of the maximum radiation (apex of the yellow triangle).

A first experiment has been performed in heading-based teleoperation mode with prior knowledge of the location of the nuclear source, which was placed in direct sight of the operator. Figure 10 shows an image of the experiment, with the UAV close to the radiating source, as well as the corresponding total accumulated spectrum. The acquired energy spectra reveals all of the main emission peaks of ^192^Ir, allowing a definitive identification of the emitting nuclear source. Figure 11 shows the total flight path of the UAV. The flight time of the experiment was about 7 min. In this experiment, the error between the actual position of the nuclear source and the location of the maximum measured radiation intensity was about 2.5 m (estimated using GPS coordinates).

**Figure 12:**
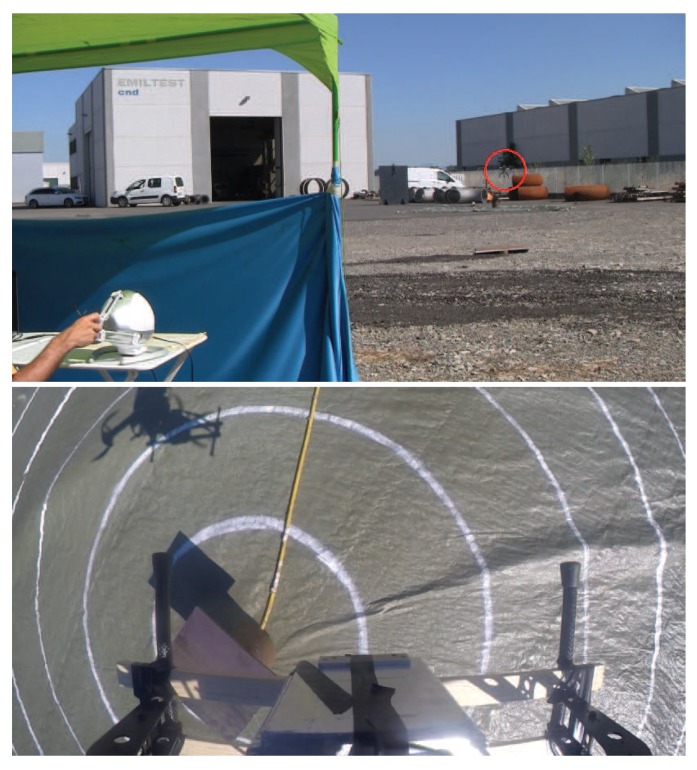
Detection of ^192^Ir (located at the center of the target within the lead container) in a real environment using the position to position teleoperation mode. Experimental setup (top), where the UAV is highlighted by the red circle. Image taken from the onboard camera (bottom) when the operator affirmed that the current position of the UAV returned the maximum measured radiation. In this set of experiments, the gimbal unit was not used to get a clearer view of the ground from the onboard camera. The low speed of the UAV ensured that the gamma-ray detector remained parallel to the ground.

To improve accuracy in measuring the error between the location of the nuclear radiation source, estimated by the operator of the haptic device, and its actual location, a video camera was installed on the UAV, pointing to the ground, to record each experiment. Indeed, the recorded videos have been used offline to measure the error with a significantly better accuracy than using the GPS sensor. A set of experiments (five repetitions) has been performed to compare the two teleoperation modes described in Section 3.1. The location of the nuclear source was changed in each experiment, and it was unknown to the operator. Indeed, the operator was sitting in front of a cloth that prevented him from seeing the nuclear source on the ground. The operator was still able to see the UAV during the task. The nuclear source was placed at the center of a target with concentric circles (0.5-m radius step size). Figure 12 shows images of the experiments. Again, each exploration experiment ended when the operator affirmed that the current position of maximum measured radiation was close enough to the nuclear source. On average, using the position to position teleoperation mode, the operator was able to detect the location of the nuclear source with a position error of 1.5±0.5 m, while using the heading-based teleoperation mode, the position error was about 2.5±0.5 m. The distance between the take-off position of the UAV and the location of the nuclear source was, on average, 20 m. The average flight time was 5 min. Experiments did not require any critical intervention by the operator of the remote controller.

### 4.3. Evaluation of Visual Feedback

**Figure 13:**
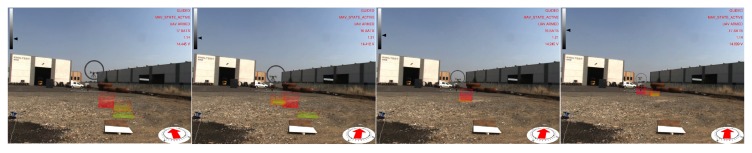
Example images of the augmented reality environment in an industrial setting.

**Figure 14:**
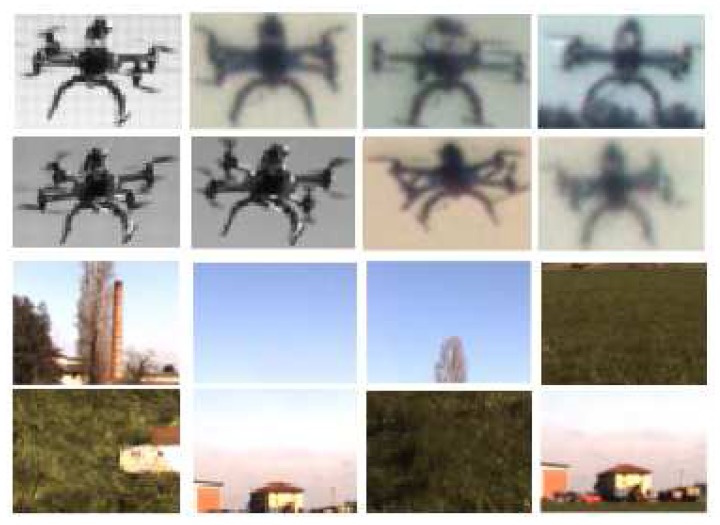
Examples of the images used in the training set for vision-based UAV detection.

**Figure 15:**
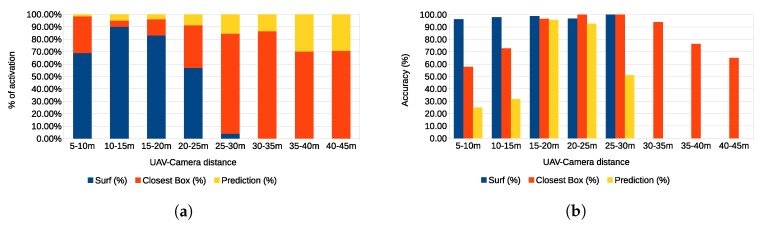
Vision-based UAV detection performance.

**Figure 16:**
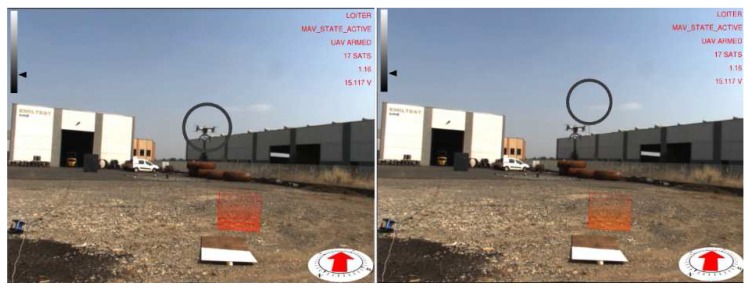
Comparison of UAV position estimation methods: vision-based (left), EKF (onboard sensors) (right). A video of the experiment is available as Supplementary Material (video2).

**Figure 17:**
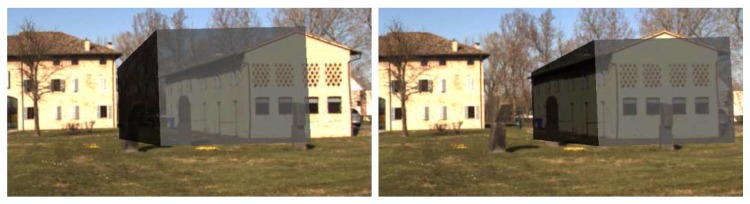
Building visualization in augmented reality: result from coarse registration (left) and after manual correction (right).

Images of the augmented reality environment are reported in Figure 13. The fixed ground camera used in the experiments (AVT Mako G125C) has a resolution of 1292×964 and a maximum frame rate of 30 fps. The camera was placed at approximately a 1.5-m height from the ground. The vision-based UAV detection algorithm takes, on average, 16.72 ms of execution time (59.8 Hz on an Intel Core i7-4720HQ). The training set (Figure 14) contained 4000 images (2000 negative samples and 2000 positive samples) acquired in a different environment (rural area). The SVM classifier, implemented by the OpenCV library, has been trained using bag-of-words with SURF features and a codebook of size 1000. The train_auto method was used, which implements a 10-fold cross-validation. The test set contained 3944 images taken from the industrial setting (Figure 13). The UAV detection algorithm was evaluated in a distance range between 5 and 45 m. Figure 15a shows the frequency of activation of the three UAV detection strategies (SURF, closest box and prediction) at different horizontal distances of the UAV to the camera. It can be noted that SURF-based estimation is mainly active when the UAV distance to the camera is under 25 m. Beyond that distance, the size of the UAV on the image reduces, so that SURF features are no longer usable; thus, closest box is mainly active. The activation frequency of the prediction-based estimation method also increases with the distance. Accuracy was computed by comparing the estimated UAV location on the image with a manually-annotated ground truth. A distance threshold of 15 pixels, estimated from an ROC curve, was used. The overall detection accuracy of the algorithm on the test set is around 90%. Figure 15b reports detailed detection results. The SURF method, when active, achieves an accuracy above 95%. The closest box method achieves an accuracy rate of about 60% for the short range (when it is rarely active) due to the fact that when the UAV is close to the camera, the search window includes spurious blobs from the ground (e.g., moving dust or grass). Closest box also achieves a 60% accuracy rate at long distances, as background subtraction can fail to detect the UAV due to its small projection on the image plane. Although the accuracy rate of the prediction method, which is mainly active at long distances, is very low, it still provides an estimated position of the UAV on the image plane that is useful to the operator to identify the UAV, as confirmed by the user study reported in Section 4.4. A comparison between the vision-based UAV detection algorithm and the result provided by the autopilot EKF is shown in Figure 16. 3D registration of buildings required a manual correction. Indeed, the coarse registration of the geographical elements based on the OSM data has an average position error of 5 m and an average orientation error of $5^{\circ }$. An example of building registration is shown in Figure 17.

### 4.4. Usability Experiments

**Figure 18:**
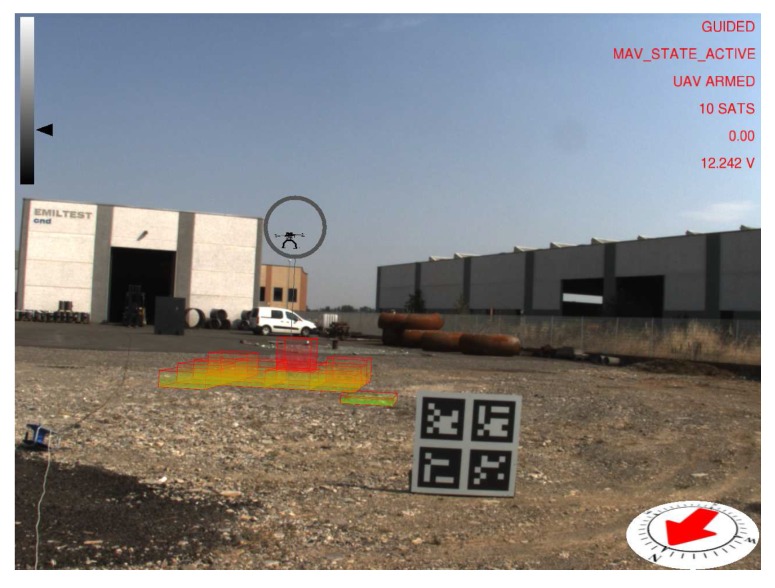
Simulated environment developed for the usability tests. The UAV is a 3D model simulated using the ArduCopter SITL simulator.

**Figure 19:**
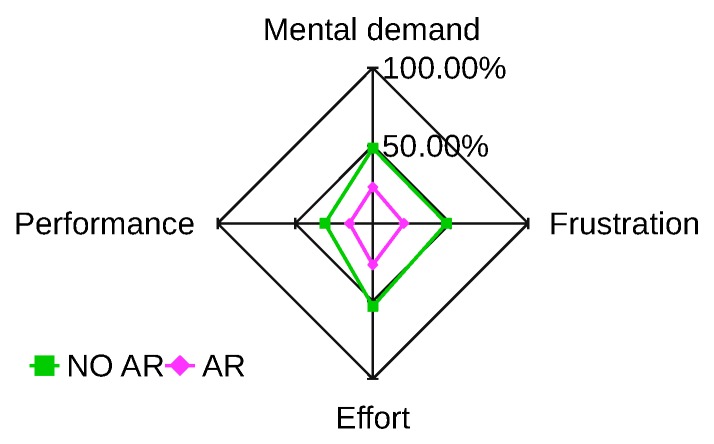
NASA-TLX questionnaire results. The AR line indicates average results of the VHAR interface; the NO AR line indicates average results without visual-feedback.

The usability of the visuo-haptic interface has been evaluated in a simulated environment. The augmented reality environment (Figure 18) displays a video stream of the real workspace, and it includes a simulated 3D model of the UAV (animated using the ArduCopter SITL simulator), as well as a simulated nuclear source. A total of 10 participants was recruited for the evaluation. Each participant, after a training session, performed two tests in random order. In one case, the user performed the nuclear radiation detection task using the visuo-haptic interface. In the other case, graphical augmentation on the video stream was disabled, and the task was performed using only haptic feedback with additional information displayed on a side screen. In particular, the side screen displayed only the radiation spectrum and a 2D plot of the UAV trajectory with a mark indicating the current location of the maximum radiation found during the mission. The evaluation was carried out from the NASA-TLX questionnaire and the SPAM (situation present assessment method) [46]. Four categories of NASA-TLX have been considered, i.e., mental demand, performance, effort and frustration (rated for each task within a 100-points range with five-point steps). High values mean high mental demand, unsuccessful performance, great effort and high frustration or stress. Figure 19 shows the results in a star-like diagram. The VHAR interface received better scores for each parameter. One-way ANOVA analysis showed that the results were statistically significant for all parameters (p≤0.05).

**Figure 20:**
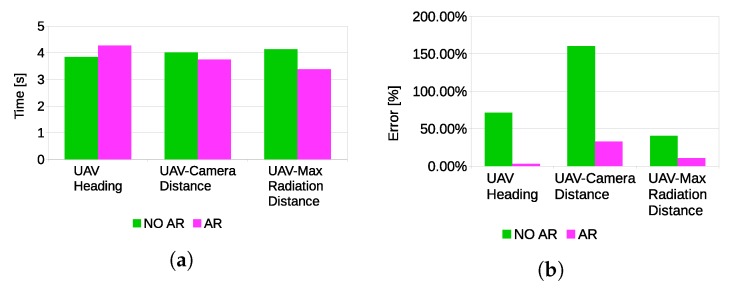
Situation present assessment method (SPAM) questionnaire results.

Participant were asked questions during the execution of the task according to the SPAM method. Questions were related to the current state of the task. In particular, three questions were asked: “What is the current heading of the UAV with respect to the magnetic north?”, “How far is the UAV from the current location of maximum radiation?”, “How far is the UAV from the camera?”. Participants explicitly stated that the visual feedback of the VHAR interface was very useful to detect the nuclear radiation source and to determine distances. Indeed, statistically-significant results (Figure 20) were found for all of the parameters estimated by the users (p≤0.05). However, the results of the questionnaire indicated no significant differences in response time. Hence, it can be concluded that the main advantage of adding visual feedback is that it can improve the situation awareness of the task. Furthermore, the results indicate that adding visual feedback did not provide a significant improvement in position detection accuracy of the nuclear source.

## 5. Conclusions

This work presented a visuo-haptic augmented reality interface for UAV teleoperation with applications to the localization of nuclear sources in outdoor environments. A lightweight CdZnTe-based gamma-ray detector was developed and installed on a UAV. Two control modes were presented, which generate an attractive force feedback around the location of the most intense detected radiation, enabling efficient exploration of potentially dangerous areas. Experiments have been carried out in outdoor environments with a real radiation source, under the supervision of the public environmental protection agency. Visuo-haptic interfaces for UAV teleoperation in hazardous environments have not been considered in previous works. Results indicate that the proposed VHAR interface increases the situation awareness of the operator and reduces mental workload. Indeed, the real-time visuo-haptic feedback provides enough information to the user to oversee the entire mission. The augmented reality environment exploits a vision-based UAV detection algorithm that achieves a high detection rate. The main limitation of the VHAR interface is that the use of a single fixed camera on the ground reduces the available field of view of the environment. Therefore, in the proposed experiments, the UAV was clearly visible to the operator for safety reasons. Additional work is needed to achieve a fully-automatic registration of geographical data that are affected by large estimation errors. The developed prototype is available to third parties (http://www.imem.cnr.it/xdrone) such as agencies for environmental control, decommissioning companies and institutions with the responsibility of providing aid in the case of disasters and accidents involving nuclear or radiological materials.

## Figures and Tables

**Table 1:** Measured radioactivity from different nuclear sources by a 20×20×6 mm detector at a 2-m distance.

Nuclear Source	Dose (mSv/year)	Source Activity (Bq)	Counts/s
Americium 241	1	$1.6\times 10^{8}$	1270
Cobalt 57	1	$5.4\times 10^{7}$	411
Cesium 137	1	$8.2\times 10^{6}$	159

## Supplementary Materials

The following are available online at http://www.mdpi.com/1424-8220/17/10/2234/s1.

## Abbreviations

The following abbreviations are used in this manuscript:

UAV	Unmanned Aerial Vehicle
VHAR	Visuo-Haptic Augmented Reality
CdZnTe	Cadmium Zinc Telluride

## References

[1] Boudergui K., Carrel F., Domenech T., Guenard N., Poli J.P., Ravet A., Schoepff V., Woo R. Development of a drone equipped with optimized sensors for nuclear and radiological risk characterization. Proceedings of the 2nd International Conference on Advancements in Nuclear Instrumentation Measurement Methods and their Applications (ANIMMA).

[2] MacFarlane J., Payton O., Keatley A., Scott G., Pullin H., Crane R., Smilion M., Popescu I., Curlea V., Scott T. (2014). Lightweight aerial vehicles for monitoring assessment and mapping of radiation anomalies. J. Environ. Radioact..

[3] Martin P., Payton O., Fardoulis J., Richards D., Scott T. (2015). The use of unmanned aerial systems for the mapping of legacy uranium mines. J. Environ. Radioact..

[4] Martin P., Payton O., Fardoulis J., Richards D., Yamashiki Y., Scott T. (2016). Low altitude unmanned aerial vehicle for characterising remediation effectiveness following the FDNPP accident. J. Environ. Radioact..

[5] Pöllänen R., Toivonen H., Peräjärvi K., Karhunen T., Ilander T., Lehtinen J., Rintala K., Katajainen T., Niemelä J., Juusela M. (2009). Radiation surveillance using an unmanned aerial vehicle. Appl. Radiat. Isotopes.

[6] Han J., Xu Y., Di L., Chen Y. (2013). Low-cost Multi-UAV Technologies for Contour Mapping of Nuclear Radiation Field. J. Intell. Robot. Syst..

[7] Neumann P., Bartholmai M., Schiller J., Wiggerich B., Manolov M. Micro-drone for the characterization and self-optimizing search of hazardous gaseous substance sources: A new approach to determine wind speed and direction. Proceedings of the IEEE International Workshop on Robotic and Sensors Environments (ROSE).

[8] Newaz A.A.R., Jeong S., Lee H., Ryu H., Chong N.Y., Mason M.T. Fast radiation mapping and multiple source localization using topographic contour map and incremental density estimation. Proceedings of the IEEE International Conference on Robotics and Automation (ICRA).

[9] Okuyama S., Torii T., Suzuki A., Shibuya M., Miyazaki N. (2008). A Remote Radiation Monitoring System Using an Autonomous Unmanned Helicopter for Nuclear Emergencies. J. Nucl. Sci. Technol..

[10] Micconi G., Aleotti J., Caselli S. Evaluation of a Haptic Interface for UAV Teleoperation in Detection of Radiation Sources. Proceedings of the 18th IEEE Mediterranean Electrotechnical Conference (MELECON).

[11] Micconi G., Aleotti J., Caselli S., Benassi G., Zambelli N., Zappettini A. Haptic Guided UAV for Detection of Radiation Sources in Outdoor Environments. Proceedings of the 3rd RED-UAS 2015 Workshop on Research, Education and Development of Unmanned Aerial Systems.

[12] Lu Y., Macias D., Dean Z.S., Kreger N.R., Wong P.K. (2015). A UAV-Mounted Whole Cell Biosensor System for Environmental Monitoring Applications. IEEE Trans. NanoBiosci..

[13] Kurvinen K., Smolander P., Pöllänen R., Kuukankorpi S., Kettunen M., Lyytinen J. (2005). Design of a radiation surveillance unit for an unmanned aerial vehicle. J. Environ. Radioact..

[14] Sanada Y., Torii T. (2015). Aerial radiation monitoring around the Fukushima Dai-ichi nuclear power plant using an unmanned helicopter. J. Environ. Radioact..

[15] Frew E.W., Brown T.X. (2009). Networking Issues for Small Unmanned Aircraft Systems. J. Intell. Robot. Syst..

[16] Towler J., Krawiec B., Kochersberger K. (2012). Radiation Mapping in Post-Disaster Environments Using an Autonomous Helicopter. Remote Sens..

[17] Zollmann S., Hoppe C., Kluckner S., Poglitsch C., Bischof H., Reitmayr G. (2014). Augmented Reality for Construction Site Monitoring and Documentation. Proc. IEEE.

[18] Sun M., Dong N., Jiang C., Ren X., Liu L. Real-Time MUAV Video Augmentation with Geo-information for Remote Monitoring. Proceedings of the Fifth International Conference on Geo-Information Technologies for Natural Disaster Management (GiT4NDM).

[19] Okura F., Kanbara M., Yokoya N. Augmented telepresence using autopilot airship and omni-directional camera. Proceedings of the IEEE Intermational Symposium on Mixed and Augmented Reality (ISMAR).

[20] Iwaneczko P., Jȩdrasiak K., Nawrat A., Nawrat A., Jȩdrasiak K. (2016). Augmented Reality in UAVs Applications. Innovative Simulation Systems.

[21] Ai Z., Livingston M.A., Moskowitz I.S. Real-time unmanned aerial vehicle 3D environment exploration in a mixed reality environment. Proceedings of the International Conference on Unmanned Aircraft Systems (ICUAS).

[22] Zollmann S., Hoppe C., Langlotz T., Reitmayr G. (2014). FlyAR: Augmented Reality Supported Micro Aerial Vehicle Navigation. IEEE Trans. Visualization Comput. Graph..

[23] Reyes S., Romero H., Salazar S., Lozano R., Santos O. Outdoor haptic teleoperation of a hexarotor UAV. Proceedings of the International Conference on Unmanned Aircraft Systems (ICUAS).

[24] Kanso A., Elhajj I.H., Shammas E., Asmar D. Enhanced teleoperation of UAVs with haptic feedback. Proceedings of the IEEE International Conference on Advanced Intelligent Mechatronics (AIM).

[25] Lam T., Boschloo H., Mulder M., van Paassen M. (2009). Artificial Force Field for Haptic Feedback in UAV Teleoperation. IEEE Trans. Syst. Man Cybern. Part A Syst. Hum..

[26] Carloni R., Lippiello V., D’Auria M., Fumagalli M., Mersha A., Stramigioli S., Siciliano B. (2013). Robot Vision: Obstacle-Avoidance Techniques for Unmanned Aerial Vehicles. IEEE Robot. Autom. Mag..

[27] Omari S., Hua M.D., Ducard G., Hamel T. Bilateral haptic teleoperation of VTOL UAVs. Proceedings of the IEEE International Conference on Robotics and Automation (ICRA).

[28] Masone C., Giordano P., Bulthoff H., Franchi A. Semi-autonomous trajectory generation for mobile robots with integral haptic shared control. Proceedings of the IEEE International Conference on Robotics and Automation (ICRA).

[29] Hou X., Mahony R., Schill F. (2015). Comparative Study of Haptic Interfaces for Bilateral Teleoperation of VTOL Aerial Robots. IEEE Trans. Syst. Man Cybern. Syst..

[30] Hou X., Mahony R., Schill F. Representation of vehicle dynamics in haptic teleoperation of aerial robots. Proceedings of the IEEE International Conference on Robotics and Automation (ICRA).

[31] Son H.I., Kim J., Chuang L., Franchi A., Giordano P., Lee D., Bulthoff H. An evaluation of haptic cues on the tele-operator’s perceptual awareness of multiple UAVs’ environments. Proceedings of the IEEE World Haptics Conference (WHC).

[32] Ruesch A., Mersha A., Stramigioli S., Carloni R. Kinetic scrolling-based position mapping for haptic teleoperation of unmanned aerial vehicles. Proceedings of the IEEE International Conference on Robotics and Automation (ICRA).

[33] Stramigioli S., Mahony R., Corke P. A novel approach to haptic tele-operation of aerial robot vehicles. Proceedings of the IEEE International Conference on Robotics and Automation (ICRA).

[34] Mersha A., Stramigioli S., Carloni R. (2014). On Bilateral Teleoperation of Aerial Robots. IEEE Trans. Robot..

[35] Mersha A., Hou X., Mahony R., Stramigioli S., Corke P., Carloni R. Intercontinental haptic teleoperation of a flying vehicle: A step towards real-time applications. Proceedings of the IEEE/RSJ International Conference on Intelligent Robots and Systems (IROS).

[36] Stegagno P., Basile M., Bulthoff H., Franchi A. A semi-autonomous UAV platform for indoor remote operation with visual and haptic feedback. Proceedings of the IEEE International Conference on Robotics and Automation (ICRA).

[37] Del Sordo S., Abbene L., Caroli E., Mancini A., Zappettini A., Ubertini P. (2009). Progress in the development of CdTe and CdZnTe semiconductor radiation detectors for astrophysical and medical applications. Sensors.

[38] Camarda G., Bolotnikov A., Cui Y., Hossain A., Kohman K., James R. CdZnTe room-temperature semiconductor gamma-ray detector for national-security applications. Proceedings of the 2007 IEEE Long Island Systems, Applications and Technology Conference, LISAT.

[39] Kowatari M., Kubota T., Shibahara Y., Fujii T., Fukutani S., Takamiya K., Mizuno S., Yamana H. (2015). Application of a CZT detector to in situ environmental radioactivity measurement in the Fukushima area. Radiat. Prot. Dosim..

[40] Kuvvetli I., Budtz-Jørgensen C., Zappettini A., Zambelli N., Benassi G., Kalemci E., Caroli E., Stephen J., Auricchio N. (2014). A 3D CZT high resolution detector for X- and gamma-ray astronomy. SPIE Int. Soc. Opt. Eng..

[41] Marchini L., Zappettini A., Gombia E., Mosca R., Lanata M., Pavesi M. (2009). Study of surface treatment effects on the metal-CdZnTe interface. IEEE Trans. Nucl. Sci..

[42] Zivkovic Z., van der Heijden F. (2006). Efficient adaptive density estimation per image pixel for the task of background subtraction. Pattern Recognit. Lett..

[43] Sourimant G., Morin L., Bouatouch K. GPS, GIS and Video Registration for Building Reconstruction. Proceedings of the IEEE International Conference on Image Processing.

[44] Karlekar J., Zhou S.Z., Nakayama Y., Lu W., Loh Z.C., Hii D. Model-based localization and drift-free user tracking for outdoor augmented reality. Proceedings of the IEEE International Conference on Multimedia and Expo (ICME).

[45] Min S., Lei L., Wei H., Xiang R. Interactive registration for Augmented Reality GIS. Proceedings of the International Conference on Computer Vision in Remote Sensing (CVRS).

[46] Durso F.T., Dattel A.R. (2004). SPAM: The real-time assessment of SA. A Cognitive Approach to Situation Awareness: Theory and Application.

